# Immunopathogenesis of Ocular Behçet's Disease

**DOI:** 10.1155/2014/653539

**Published:** 2014-07-02

**Authors:** Un Chul Park, Tae Wan Kim, Hyeong Gon Yu

**Affiliations:** ^1^Department of Ophthalmology, National Medical Center, 245 Euljiro, Jung-gu, Seoul 100-799, Republic of Korea; ^2^Department of Ophthalmology, Seoul Metropolitan Government Seoul National University Boramae Medical Center, No. 395 Shindaebang-2-dong, Dongjak-gu, Seoul 156-707, Republic of Korea; ^3^Department of Ophthalmology, Seoul National University College of Medicine, 103 Daehak-ro, Jongno-gu, Seoul 110-799, Republic of Korea; ^4^Sensory Organs Institute, Medical Research Center, Seoul National University, 103 Daehak-ro, Jongno-gu, Seoul 110-799, Republic of Korea

## Abstract

Behçet's disease (BD) is a chronic recurrent systemic inflammatory disorder of unknown etiology characterized by oral and genital ulcerations, skin lesions, and uveitis. The ocular involvement of BD, or Behçet's uveitis (BU), is characterized by panuveitis or posterior uveitis with occlusive retinal vasculitis and tends to be more recurrent and sight threatening than other endogenous autoimmune uveitides, despite aggressive immunosuppression. Although pathogenesis of BD is unclear, researches have revealed that immunological aberrations may be the cornerstone of BD development. General hypothesis of BD pathogenesis is that inflammatory response is initiated by infectious agents or autoantigens in patients with predisposing genetic factors and perpetuated by both innate and acquired immunity. In addition, a network of immune mediators plays a substantial role in the inflammatory cascade. Recently, we found that the immunopathogenesis of BU is distinct from other autoimmune uveitides regarding intraocular effector cell profiles, maturation markers of dendritic cells, and the cytokine/chemokine environment. In addition, accumulating evidence indicates the involvement of Th17 cells in BD and BU. Recent studies on genetics and biologics therapies in refractory BU also support the immunological association with the pathogenesis of BU. In this review, we provide an overview of novel findings regarding the immunopathogenesis of BU.

## 1. Introduction

Endogenous autoimmune uveitis comprises a clinically heterogeneous group of intraocular inflammatory diseases of various types and etiologies that can lead to blindness [1]. It is thought to be triggered by various specific and nonspecific agents in the early disease stage, but the chronicity of the inflammatory process is influenced by endogenous host factors, in which the immune system plays an important role. Although autoimmune uveitis includes a range of clinical entities (most of idiopathic origin), its immunological findings are characterized by the predominant infiltration of T cells, which implies that endogenous uveitis is a T cell-mediated autoimmune disease [2]. The development of autoimmune uveitis depends on several factors such as the nature of antigenic stimulus, subsets of competent antigen-presenting cells (APCs), increased chemotaxis of inflammatory cells, and related inflammatory mediators (e.g., chemokines and cytokines) produced by these cells.

Behçet's disease (BD) is a chronic, recurrent systemic inflammatory disorder characterized by oral and genital mucous ulcerations, skin lesions, and uveitis. The pathogenesis of BD remains unclear, but microbial triggers, environmental factors, endothelial dysfunction, genetic predisposition, and immunological abnormalities have been implicated [3]. Behçet's uveitis (BU) is characterized by chronic panuveitis or posterior uveitis with necrotizing retinal vasculitis and tends to be more recurrent and sight threatening than other endogenous uveitides such as Vogt-Koyanagi-Harada (VKH) disease or HLA-B27-associated uveitis. Previously, in a series of reports, we showed that immunopathogenic mechanism of BU probably differs from those of other endogenous uveitides [4–7]. In BD patients with active uveitis, immune effectors in aqueous humor and peripheral blood differentiated the disease from endogenous uveitis of other origins [4, 5]. The intraocular cytokine environment and chemokine expressions in intraocular lymphocytes were also different between active BU and non-Behçet's uveitis [6, 7]. Higher expressions of maturation markers in dendritic cells (DCs) may reflect disease activity in BU [8]. Furthermore, these factors can contribute to the chronic and recurrent nature of BU. In this paper, we review advances in the immunopathogenesis of BU with regard to antigens, immune cells and mediators, genetics, and immune therapy with biologics.

## 2. Antigens and Autoantigens in the Pathogenesis of Behçet's Uveitis

Oral ulcers are the first symptom in about 70% of BD patients, and decrease of some disease symptoms after antibiotic treatment suggests a role of bacteria in the etiology of BD [9, 10]. In addition, the HSV-1 genome and serum antibodies against the virus have been reported in a higher proportion of patients with BD than in normal controls [9]. Moreover, the inoculation of HSV into mice was found to cause ocular inflammation mimicking BU [11]. Some bacteria such as* Borrelia burgdorferi* and* Helicobacter* have been proposed to act as triggering factors in BD, but no evidence has been presented that BD is a result of direct infection by viruses or bacteria.

**Figure 1:**
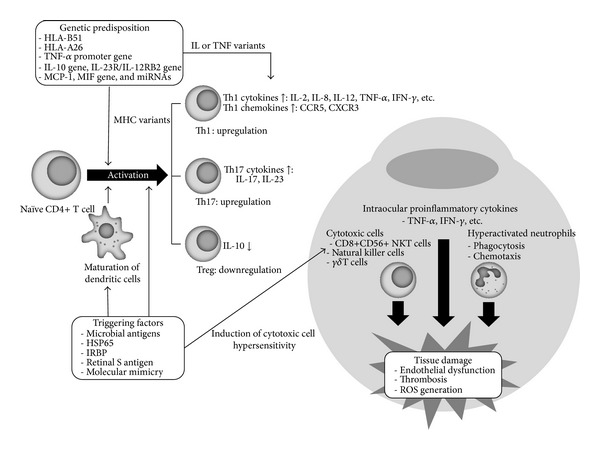
Immunopathogenesis of Behçet's uveitis. Hypersensitivity of T cells and cytotoxic cells to various antigens and predisposing genetic factors play a crucial role in the pathogenesis of Behçet's uveitis. The high maturation profiles of dendritic cells can contribute to the perpetuation of the inflammation, and chemokines and cytokines are mediators that can generate and augment the immune response in inflammatory cascade.

It has been proposed that cross-reactivity between microbial heat shock protein (HSP) and human HSP underlies the relation between infection and autoimmunity [12] (Figure 1). Some peptides within mycobacterial 65 kDa HSP sharing significant homology with those of human mitochondrial HSP have been demonstrated to be responsible for the proliferation of *γ* *δ* T cells in BD patients [13, 14]. In this regard, Direskeneli and Saruhan-Direskeneli [15] proposed a role for HSP65 as a potential T cell antigen.

T cell responses against retinal autoantigens have been demonstrated in various types of ocular inflammations such as BU. Some epitopes of S antigen were found to share homology with particular amino acid regions of HLA-B51 and HLA-B27, which suggests that this antigen contributes to the pathogenesis of BU [16, 17]. In addition, retinal tissue damage and significant increase in nitric oxide (NO) production were found in experimental models of autoimmune uveitis induced by S antigen or interphotoreceptor retinoid binding protein [18, 19].

## 3. Immune Cells in the Pathogenesis of Behçet's Uveitis

### 3.1. Antigen-Specific Effector T Cells as Inducers

CD4+ T cells play an important role in immune system by orchestrating the function of other immune cells. When activated by pathogen, naïve CD4+ T cells differentiate into two different functional subsets, that is, helper T cell type 1 (Th1) and type 2 (Th2), which differ in terms of cytokine production. Th1 cells produce the cytokines interferon-gamma (IFN-*γ*) and tumor necrosis factor-alpha (TNF-*α*), which activate macrophages and are responsible for cell-mediated immunity. In contrast, Th2 cells produce cytokines such as interleukin-4 (IL-4) and IL-13 which are responsible for antibody-mediated immunity by B cells [20]. Th1 and Th2 cells play important regulatory roles in the immune system [21], and Th1/Th2 imbalance may underlie the pathogenesis of several autoimmune diseases [22].

Previously, our analysis of immune cell types in active BU revealed that the predominant intraocular infiltrating cells were CD8+ T cells, whereas CD4+ T cells were the predominant infiltrating cells in patients with other uveitides [4]. In particular, in BU, the number of CD3+CD56+ cells (NKT cells) was much higher in aqueous humor than in other uveitides, and CD8+CD56+ cells were the predominant subtype among NKT cells. These unique features displayed by the cytotoxic effectors of BU may reflect the more recurrent and greater destructive nature of BU than of other uveitides. Furthermore, immune reactions evoked by certain infectious agents or by the autoantigens presented by APCs might induce the immunopathogenic hypersensitivity of cytotoxic effector cells. Additional study by our group showed that CD8^bright^CD56+ T cells in BU patients have a cytotoxic effector phenotype with functional NK receptor and they exert cytolytic functions against vascular endothelial cells through fasL- and perforin-dependent pathways [23]. The predominance of CD8+ T cells and NKT cells in the intraocular infiltrating cell population in active BU is in line with the unique increase in aqueous IL-15 levels. In active BU patients, intraocular IL-15 level is more elevated than in other endogenous uveitides [6, 24]. IL-15 is involved in the development and survival of immune effector cells, such as NK cells, NKT cells, and CD8+ T cells, and contributes to homeostasis and to the activation of *γ* *δ* T cells [25, 26]. Thus, IL-15 is considered to participate in the selective recruitment of unique effectors in BU.

However, the mechanism underlying the hypersensitivity of T cells to various antigens has not been determined, and, presently, it is unclear whether this hypersensitivity is due to intrinsic T cell defects or whether it occurs secondary to functional abnormalities of DCs.

### 3.2. Roles of Dendritic Cells in Behçet's Uveitis

DCs are the most potent APCs and play a crucial role in the polarization of naïve T cells into Th1 or Th2 cells [27]. DCs mature during their migration from the periphery to lymph nodes, and this process involves the upregulations of major histocompatibility complex (MHC) class II and costimulatory molecules [28–33].

We have shown that peripheral blood DC maturation profiles in patients with endogenous uveitis including BU exhibit higher expression of MHC class II and costimulatory molecules even in the absence of uveitis compared with healthy controls. This suggests that the maturation status of DCs is important in perpetuating inflammation as well as generating uveitis [8]. The relatively high expression of costimulatory molecules and MHC class II in BD patients in remission suggests that DC maturation is related to the chronicity and recurrence of uveitis. DCs may be involved in the continuous activation of naïve or central memory cells in lymph nodes draining inflammatory sites. In addition, recent reports have suggested that immature and mature DCs have different functions and that immature DCs have a potential therapeutic role in other autoimmune diseases [34, 35].

Epidermal Langerhans cell counts are significantly elevated at sites of pathergy reactions [36], and these cells are more active in BD patients than in healthy controls [37]. Pay et al. [38] performed phenotypical analysis of DC subsets in peripheral blood and found that BD patients had lower plasmacytoid DC percentages than healthy controls. This result indicates the probable migration of these cells and their accumulation in inflamed tissues and suggests active participation of this cellular subset in the pathogenesis of BD.

These observations may be taken as an evidence of DC functional abnormality in BD, but it is not fully understood whether these functional abnormalities are due to a primary defect or occur secondary to deviant interactions between immune cells during antigen presentation. Further studies are required to explain the pathogenic role of APCs, rather than T cells, in BD.

### 3.3. Roles of Neutrophils in Behçet's Uveitis

Neutrophils constitute the major component of the innate immunity system. Neutrophil hyperactivation, which can be confirmed by the upregulations of CD10, CD11a, and CD14 on cell surfaces, is believed to play an important role in the pathogenesis of BD by increasing chemotaxis, phagocytosis, superoxide generation, and myeloperoxidase levels [39–42]. Although mechanism of hyperactivation is not fully understood in BD, proinflammatory cytokines secreted by T cells or APCs such as TNF-*α*, IL-8, IFN-*γ*, or G-CSF appear to be responsible for the priming of neutrophils [43, 44]. In addition to Th1 cytokines, Th17 cytokine, IL-17, has been reported to promote neutrophil recruitment to sites of inflammation and to regulate neutrophil-associated inflammation [45, 46]. Furthermore, inflammatory cytokines produced by activated neutrophils, such as IL-18, seem to promote neutrophil activation, and in so doing they create a vicious cycle [43, 47].

Histopathological analyses of BD inflammatory lesions have shown neutrophils which represent the major cell population in vascular infiltrates [40, 48]. Furthermore, the generation of reactive oxygen species by activated neutrophils has been reported to be elevated in BD patients [49, 50] and thought to be responsible for tissue damage by causing vascular endothelial cell dysfunction and thrombosis [51–53].

In recurrent uveitis attacks in BD, hypopyon consists of inflammatory cells infiltrating the eye, most of which are polymorphonuclear cells (PMNs) [54]. Fujimori et al. [39] reported fluctuations in the apoptosis level of PMNs in accord with uveitic activity in BD patients and attributed this to impairment of the FasL-Fas interaction, which the authors suggested might explain the alternation between sudden inflammation attacks and self-limiting resolution in BU. In a small case series, absorptive neutrophil apheresis in refractory ocular BD patients reduced the frequencies of ocular attacks implying that neutrophil hyperactivity plays a role in the pathogenesis of BU [55].

## 4. Cytokine and Chemokine Environment in Behçet's Uveitis

Th1 immune response plays an important role during the pathogenesis of BD. Increased productions of Th1 cytokines such as IL-2, IL-12, and IFN-*γ* have been reported in the peripheral blood mononuclear cells (PBMCs) of active BD patients, while the frequency of Th2 cytokine IL-4 producing cells was decreased [56–59]. In particular, IL-12 is primarily produced by APCs and plays a crucial role in Th1 polarization. Furthermore, Th1-polarized cytokine environment rich in IL-8, IL-12, and IL-12 mRNA, TNF-*α*, and IFN-*γ* has been reported in the active inflammatory lesions of BD patients, such as skin, oral and genital mucosa, stomach, intestine, and eye [6, 22, 60–62].

Uveitis in BD can be distinguished from other causes of uveitis by its intraocular cytokine profile as well as its immune effectors. In ocular BD patients, Th1-dominant immune response is observed both in peripheral blood and in aqueous humor as in other endogenous uveitides or in other inflammatory sites of BD patients [6, 7, 24, 61]. However, Th1 polarization in BU tends to be more extreme than that observed in endogenous uveitides of other causes and shows higher proinflammatory but lower immunosuppressive cytokine profiles [6, 24]. Previously, we reported that active BU patients have higher aqueous levels of IFN-*γ* and TNF-*α* but lower level of IL-4 than non-Behçet's uveitis patients [6]. In addition, IL-10, the immunosuppressive regulatory T cell (T_reg_) related cytokine, was not detected in any BU patient.

IFN-*γ*-producing T cell number has been reported to be elevated in active BU patients [5, 23, 59]. CD56+ T cell subsets, including CD8^bright^CD56+ and CD56+*γ* *δ* T cells, are considered to be the cell population primarily responsible for IFN-*γ* production [5], and CD8^bright^CD56+ T cells in active BD uveitis have been reported to be polarized to produce larger amounts of IFN-*γ* on stimulation than those in inactive BD and normal controls [23]. In active BU, IFN-*γ* levels are much higher in aqueous humor than in serum, which suggests that it is actively produced in eyes [5, 6]. A classical Th1 type cytokine IFN-*γ* exerts its cytotoxic effects by promoting cytotoxic CD8+ T cell-mediated target cell destruction and inducing NO production [63, 64]. In addition to the presumed fasL- and perforin-dependent pathways of CD8+CD56+ T cells, oxidative stresses such as those produced by NO-associated process are thought to be involved in pathogenesis of obliterating vasculitis, one of main features of ocular BD [49, 65]. In particular, the cytotoxic effects of IFN-*γ* and TNF-*α* on vascular endothelial cells were shown to be mediated by elevated NO production [63]. BD patients with active uveitis were reported to exhibit significantly higher levels of IFN-*γ* and NO in plasma and PBMC supernatants than inactive BU patients or controls [53, 66]. Moreover, IFN-*γ* significantly increased NO production by PBMCs from BU patients* in vitro* whereas IL-10 decreased NO production, which suggests that IL-10 has immunoregulatory function [53].

The expression of Th1-related chemokine receptors, such as CCR5 and CXCR3, is upregulated in active BD [22, 56, 60, 62], which is consistent with our finding that CXCR3 is preferentially expressed on intraocular CD8+ T cells in BU [7]. Because CXCR3 is considered a marker of the main producers of IFN-*γ* in the T cell population, CXCR3+ T cells may be the primary effectors of the maintenance of uveitis in BD [56].

## 5. Novel Helper T Cell Pathways in Behçet's Uveitis

### 5.1. Th17-Type Immune Response in Behçet's Uveitis

Although BD used to be regarded as a Th1-mediated disease, accumulating evidence suggests that both Th1 and Th17 pathways contribute to its pathogenesis via the involvements of their respective proinflammatory cytokines. Furthermore, recent studies have shown that Th17 cells, a subset of T helper cells unrelated to Th1 and Th2, are implicated in many autoimmune and inflammatory diseases [51, 67, 68]. Th17 cells regulate inflammation by promoting the productions of distinct cytokines such as IL-17, IL-21, IL-22, and IL-26 [69]. Circulating Th17 cell proportion and the ability to produce IL-17, the representative Th17 cytokine, are both enhanced in active BD, and Th17 levels decrease during the remission stage versus the active stage [70, 71]. In patients with endogenous uveitis including BU, aqueous levels of IFN-*γ* and IL-17 are significantly higher than in controls, suggesting that both Th1 and Th17 cells are involved in the pathogenesis of endogenous uveitis [24]. IL-23, another Th17-associated cytokine, is a member of the IL-12 family sharing a p40 subunit with IL-12 and is believed to play an important role in the survival and maintenance of Th17 cells [72, 73].

Recent reports suggest that Th17/Th1 and Th17/T_reg_ balances are important regulators of inflammation activity in BD [51, 70, 74–77]. More specifically, patients with active BD have a significantly higher Th17/Th1 cell ratio in peripheral blood than healthy controls, and this is more prominent in patients with folliculitis or uveitis [74]. Under inflammatory conditions, T_reg_ cells can convert into Th17 cells under the influence of IL-1*β* or IL-2 [71, 78]. On the other hand, the reduction in Th17 levels during the inflammation remission stage in BD is believed to be due to the conversion of Th17 cells to T_reg_ cells [71, 79]. In one study, the stimulation of CD4+ T cells with IL-21 increased Th17 and Th1 differentiation but decreased T_reg_ cell proportions in peripheral blood, and the inhibition of IL-21 restored Th17/T_reg_ homeostasis [80].

In BD patients with active uveitis, IL-17 levels are elevated in peripheral blood or ocular fluid [24, 79, 81, 82], and it has been established that CD4+CD45RO+ (memory) T cells and *γ* *δ* T cells are major sources of IL-17 [82–87]. Chi et al. [81, 82] showed that serum levels of IL-17, IL-23, and IFN-*γ* were significantly higher in BD patients with active uveitis than in BD patients without uveitis or healthy controls. In their study, recombinant IL-23 was found to upregulate IL-17 production, indicating that elevated IL-23 in BU patients exerts its role by enhancing IL-17 production [82]. In another study, Th17 cell lines from active BU patients exposed to anti-TNF-*α* antibody* in vitro* failed to produce IL-17 and showed diminished expression of the Th17 transcription factor, ROR*γ*t, suggesting that TNF-*α* plays a role in Th17 differentiation [79].

### 5.2. Th22-Type Immune Response in Behçet's Uveitis

Recently, another novel subset of CD4+ effector Th cells (Th22) was identified which mainly produces IL-22 and TNF-*α* but no other T helper cell marker cytokines such as IFN-*γ* (Th1), IL-4 (Th2), or IL-17 (Th17) and thus is considered to be distinct from Th1, Th2, and Th17 cell types [88, 89]. Furthermore, accumulating evidence indicates that Th22 cells are involved in pathogenesis of various autoimmune diseases such as systemic lupus erythematosus, rheumatoid arthritis, and multiple sclerosis [90–93].

IL-22 producing CD4+ cells have been reported to play a role in BD patients with active uveitis [94, 95]. Sugita et al. [94] established Th22-type T cell clones from the aqueous humor of active BU patients and found that they produced large amounts of IL-22 and TNF-*α* but no Th1 or Th17 cytokines. When exposed to infliximab* in vitro*, Th22 cells produced less Th22-related molecules, suggesting that TNF-*α* plays a role in Th22 differentiation in BD. In another study, it was found that IL-22 levels in the supernatants of stimulated PBMCs were higher for BD patients with active uveitis than for patients without uveitis or normal controls. In addition, IL-22 levels were found to be correlated with the severity of retinal vasculitis and anterior chamber inflammation [95].

## 6. Genetic Mechanisms of Susceptibility to Behçet's Uveitis

### 6.1. Genetic Predisposition to Behçet's Disease

Since the strong association between BD and HLA-B51 was found by Ohno et al. [96], many studies from different ethnic groups have confirmed their finding [97–107]. A meta-analysis of 4,800 BD patients and 16,289 controls included 78 studies that reported a pooled OR of 5.78 (95% CI 5.00–6.67) for the development of BD by HLA-B51/B5 carriers as compared with controls [108]. Furthermore, the study estimated population attributable risks of HLA-B51/B5 for BD development to be 32–52% within different geographic areas. A recent large-scale genome-wide association study (GWAS) conducted in Turkey with the largest cohort recruited to date confirmed this result with an OR of 3.49 (95% CI 2.95–4.12) [109]. Nevertheless, the role of HLA-B51 in the pathogenesis of BD remains unclear. Suggested mechanisms include the presentation of HLA-B51-restricted peptides to CD8+ T cells or interaction with NK cells, CD8+ T cells, and *γ* *δ* T cells via its HLA-Bw4 epitope, but much remains to be clarified [110–113]. Recently, two large-scale GWAS indicated an association between BD and MHC class I complex near the HLA-A gene, which was suggested to be HLA-A26 by another GWAS conducted in Japan, independent of HLA-B51 [109, 114, 115]. This association has been observed in other populations [116–118].

Regarding MHC genes other than HLA-B51 and HLA-A, associations have been reported between HLA-B15 [119], HLA-B27 [120], and HLA-B5701 [121] and BD, but these associations have not been confirmed by GWAS. MIC-A (MHC class I chain-related gene A) was also considered to be responsible for BD susceptibility, because its location is just 46 kb centromeric to HLA-B [122–124]. But recent data suggest that the association between BD and MIC-A depends on the real disease susceptibility of HLA-B51, as MIC-A and HLA-B51 are in strong linkage disequilibrium [115, 125–127]. The TNF gene is located in the HLA class III region, and there have been discrepancies in the association between TNF-*α* promoter polymorphisms and BD [128–131]. A recent meta-analysis revealed a significant association between BD and TNF-*α* polymorphisms (−238A, −857T, and −1031C) [132], but GWAS failed to confirm these associations [109, 114].

Beyond the MHC, several genes encoding for cytokines, chemokines, or immunoregulatory proteins have been assessed with respect to their participations in the pathogenesis of BD, but results obtained were inconsistent [51, 133, 134]. Two recent GWAS reports from Turkey and Japan and their additional meta-analyses which included cohorts from Europe, Middle East, and Korea revealed consistent and significant associations between the IL-10 and IL-23R/IL-12RB2 genes and BD [109, 114]. In particular, the IL-10 gene variant was associated with reduced mRNA expression and IL-10 production [109]. IL-10 is a major anti-inflammatory cytokine and downregulator of Th1 immune response, and in the context of ocular inflammation, IL-10 plays a role in the development of anterior chamber-associated immune deviation via the induction of T_reg_ cells and inhibition of Th1 response [135]. Other studies have provided evidence that promoter region polymorphisms or haplotype-tagging polymorphisms in the IL-10 gene are associated with ocular involvement of BD [136, 137]. IL-23, which shares p40 subunit with IL-12 as described above, is a proinflammatory cytokine that promotes the Th17 pathway and its association with BD implies the importance of Th17 response in BD pathogenesis.

### 6.2. Genetic Studies on Behçet's Uveitis

In view of the high rate of ocular involvement in BD patients, identified genetic susceptibility factors to BD might exert their effects on the pathogenesis of uveitis in BD in the same manner. However, genetic studies solely for BD patients with uveitis could provide further understanding of the pathogenesis of BU, although there have been limited numbers of such studies.

Recently, case control association studies in Chinese Han population showed that monocyte chemoattractant protein- (MCP-) 1 gene and migration inhibitory factor (MIF) gene polymorphisms were associated with ocular BD [138, 139]. MCP-1, now known as CCL2, is a potent chemokine that contributes to monocyte recruitment during infection or inflammation, [140] and MIF is an important regulator of innate immunity that promotes the proinflammatory functions of immune cells [141]. In BU patients, serum levels of both proteins have been reported to be elevated [142, 143], and this suggests that the MCP-1 (CCL2) and MIF genes contribute to genetic predisposition to BU.

MicroRNAs (miRNAs) have been recently shown to be important regulators of immune homeostasis [144, 145], and two studies have reported association between miRNA and ocular BD. In one study, miR-155 expressions in PBMCs and DCs from BD patients with active uveitis were downregulated but not in VKH disease patients with active uveitis as compared with healthy controls [146]. A further* in vitro* study showed that miR-155 negatively regulates the productions of proinflammatory cytokines and the intracellular IL-17 expression of CD4+ T cells, suggesting the relevance of miR-155 downregulation in the pathogenesis of BD. More recently, the miR-146a polymorphism was reported to show a strong association with BD [147]. In this study, the CC genotype and the C allele of rs2910164 were protective against BU, and the CC genotype was associated with lower production of proinflammatory cytokines including IL-17, TNF-*α*, and IL-1*β* by PBMCs.

## 7. Immunologic Relevance of Biologic Agents in Behçet's Uveitis

Although conventional anti-inflammatory and immunosuppressive therapy are effective in most uveitis entities, they are sometimes unsuccessful in some refractory uveitis such as BD. In addition, conventional therapies act nonspecifically and can sometimes result in systemic adverse effects. Cytokines are being increasingly recognized as critical mediators of autoimmune uveitis, and treatments with biologics that interfere with immunological pathways or relevant cytokines have shown therapeutic efficacy in noninfectious uveitis including BU [148–151].

### 7.1. Anti-TNF-*α* Therapy for Behçet's Uveitis

TNF-*α* is a pleiotropic cytokine that plays a major role in pathogenesis of various inflammatory disorders and autoimmune diseases such as noninfectious uveitis. TNF-*α* exerts its proinflammatory effects by activating macrophages, facilitating CD4+ T cell development, and upregulating other cytokines, and its levels have been shown to be elevated in the serum and ocular fluid of uveitis patients, especially during the active phase [6, 152, 153]. Experimentally, lower levels of tissue damage and fewer activated macrophages and PMNs were found in retina after inhibiting TNF-*α* [154].

Infliximab, a human-murine chimeric monoclonal antibody against TNF-*α*, was the first anti-TNF-*α* agent used to treat BU [155]. In 2006, an expert panel on BD recommended that a single infusion of infliximab could be used as a first-line therapy to achieve rapid response in cases with posterior uveitis and significant visual impairment of less than 20/100, inflammation of the macular area, and bilateral posterior inflammation [156]. After this recommendation was made, a number of reports provided evidence regarding the efficacy of infliximab for the treatment of BU [157–162]. A prospective comparative study showed that infliximab enabled faster and more efficient inflammation control than intravenous or intravitreal corticosteroids in BD patients with panuveitis [162]. In BD patients, a single infusion of infliximab significantly reduced the number of TNF-*α*-secreting PBMCs within 24 hours [163]. Recently, the intravitreal use of infliximab in BU was reported to provide intraocular inflammation control without adverse effects [164].

Although the mechanism responsible for the effect of anti-TNF-*α* agents has not been elucidated, the influence of infliximab on T cell dysregulation in BD has been investigated. CD4+ T cells obtained from peripheral blood of infliximab-treated patients with refractory uveitis including BU showed higher expression of the T_reg_-specific marker Foxp3 than patients treated with colchicine or cyclosporine, suggesting the usefulness of infliximab in uveitic patients with decreased peripheral T_reg_ cell counts [165]. In addition, infliximab was found to suppress the* in vivo* and* in vitro* expansion and activation of *γ* *δ* T cells, which have potent cytotoxic effector activity [166]. Recently, gene expression profiles in PBMCs from refractory BU patients were investigated using DNA microarray technology, and infliximab treatment was found to reduce the expression of inflammatory cytokine-related genes such as IL-2R, IFN-*γ*R, IL-6, IL-6R, gp130, and IL-17R [167].

### 7.2. Interferon Therapy for Behçet's Uveitis

IFN-*α* is a naturally occurring cytokine, which is produced in response to viral infection mainly by plasmacytoid DCs [168]. It has been shown that plasmacytoid DCs obtained from patients with refractory panuveitis including BU showed reduced capacity to produce IFN-*α* after stimulation, which implies that IFN-*α* therapy could augment the defective function of plasmacytoid DCs in these patients [169].

Since its first use for the treatment of BD in the 1980s, a number of publications have reported on the beneficial effect of IFN-*α*, usually human recombinant IFN-*α*-2a, in recalcitrant forms of BU [170–178]. Although dose regimens varied, response rates (partial or complete) ranged between 78% and 98% with good functional outcomes and diminished uveitis attack frequencies. The antiproliferative and proapoptotic effects of IFN-*α* have been well established [179], but the therapeutic efficacy of IFN-*α* in refractory uveitis in BD might be due to its immunomodulatory effect. Although much remains to be elucidated, suggested immunomodulatory mechanisms include the activation of immature DCs [168], the stimulation of B cells [168], the normalization of *γ* *δ* T cell number [180], the induction of T_reg_ cells or NK cells [169, 181], and elevated levels of soluble adhesion molecules [182, 183].

### 7.3. Biologic Agents against Other Target Molecules (Interleukin and CD20)

Interleukins are a group of cytokines that are critically required by the immune system. They convey information between leukocytes and promote the activation, differentiation, proliferation, and regulation of cells. Thus, treatments that interrupt the pathways of proinflammatory interleukins could provide effective inflammation control. To date, several interleukin inhibitors against Th1 and Th17 response interleukins, such as antibodies to IL-1*β* (gevokizumab), IL-1R (anakinra), IL-2R (daclizumab), IL-6R (tocilizumab), and IL-17A (AIN457, secukinumab), have been demonstrated to be effective in refractory noninfectious uveitis [184–189]. However, their use in BU is only supported by limited evidence. Gevokizumab has been reported to provide rapid and sustained control of inflammation in BU patients resistant to conventional immunosuppression [185]. AIN457 (secukinumab) was found to have therapeutic effect in chronic noninfectious uveitis including BU, which implies Th17 immune response features in the pathogenesis of BU [189]. On the other hand, daclizumab, an IL-2R antagonist, was not found to be effective in BU in a randomized controlled trial [190], though beneficial in other forms of noninfectious uveitis [186, 187]. We lack information on the therapeutic effects of other anti-interleukin agents in BU.

CD20 is a surface antigen expressed on early to mature B cells and is another immunotherapy target molecule. Rituximab, a monoclonal anti-CD20 antibody, is an effective treatment for systemic autoimmune diseases [191], and it appears that rituximab has cytotoxic effects on B cells due to complement-mediated cell lysis or cell-mediated cytotoxicity [192]. Recently, rituximab has been reported to be efficient for inflammation control in intractable BU, but more evidence is needed [193, 194].

## 8. Conclusion

Advances in immunological and genetic studies have broadened our understanding of the immunopathogenesis of BD, but much remains uncertain. Recent evidence shows derangement of T cell homeostasis; for example, the upregulations of the Th1 and Th17 pathways and decreased inhibitory regulation by T_reg_ cells play key roles in the pathogenesis of BD. In particular, BD patients with active uveitis exhibit unique intraocular cytokine/chemokine environment and cytotoxic effector cell profiles, which imply that they influence the ocular manifestations characterized by recurrent and chronic inflammation. Furthermore, the favorable therapeutic effects of biologics in refractory BU and growing evidence of genetic susceptibility to BD suggest the importance of immune system in the pathogenesis of ocular BD.

Research into the immunopathogenic processes involved in the development of BU could define critical points in the induction of ocular inflammation and open new possible means of rational therapeutic intervention as well as customized treatment in each BU patient.
